# University students’ subjective experiences with problem-based learning and associated generic skills

**DOI:** 10.3389/fpsyg.2025.1618997

**Published:** 2025-07-29

**Authors:** Ahram Lee, Eunju Jung

**Affiliations:** ^1^Graduate School of Education, Korea University, Seoul, Republic of Korea; ^2^Department of Education, Sejong University, Seoul, Republic of Korea

**Keywords:** problem-based learning, higher education, university students, learning experience, generic skills, document analysis

## Abstract

**Introduction:**

With the rapid change in society and advancements in technology, higher education needs to focus on cultivating students’ generic skills to foster adaptability in this ever-evolving society. Problem-based learning (PBL) is acknowledged as an effective approach to engage students in self-directed, real-life-based learning experiences that enhance generic skills. The current study investigated how university students subjectively experienced PBL and explored generic skills associated with their learning.

**Methods:**

University students’ reflection papers were used as document data for analysis. A total of 58 reflection papers were collected from three PBL courses offered at a 4-year university in Seoul, Korea, between 2022 and 2023. Students were provided with open-ended questions that prompted them to give detailed descriptions of their learning experiences during PBL. The content of the papers was analyzed using inductive thematic analysis

**Findings:**

The analysis revealed four key themes related to students’ experiences with PBL: collaboration within the team, personal growth, higher-order thinking, and connection to the real world. Each theme included three sub-themes, and these themes were discussed in relation to associated generic skills.

**Discussion:**

The findings offer insights into students’ experiences during the PBL process and highlight how these experiences foster the development of cross-cutting skills applicable to various domains of life. This study extends the existing body of research, which has largely focused on quantitative measures of specific competency changes through PBL, by inductively exploring students’ subjective experiences and deriving relevant generic skills.

## 1 Introduction

The rapidly changing society and the development of technology have changed the landscape of job markets, and higher education must foster the next generation who can effectively navigate the future society with innovation (Hoidn and Kärkkäinen, 2014). Innovation requires certain generic skills, also referred to as transferable skills or core competencies, that are domain-general, such as thinking skills, social skills, and behavioral skills that can be flexibly applied in different contexts (Barrie, 2006; Hoidn and Kärkkäinen, 2014; Virtanen and Tynjälä, 2019), and fostering generic skills is related to the employability of graduates in higher education (Sarkar et al., 2020). Thus, higher education needs to cultivate students’ generic skills to help them be prepared for the adaptive transition from school to the workplace.

In South Korea, the Korea Collegiate Essential Skills Assessment (K-CESA) was first developed in 2006 by the Ministry of Education and the Korea Research Institute for Vocational Education and Training to assess college students’ generic skills: communication skills; global skills; interpersonal skills; comprehensive thinking skills; resource, information, and technology utilization skills; and self-management skills (K-CESA, 2024). Building on these basic skills, each university has established its own set of core skills and implemented competency-based education to foster competitive graduates for the future job market (Jin et al., 2011).

One of the teaching methods used in higher education that can enhance students’ competencies is problem-based learning (PBL). PBL refers to a student-centered, self-directed, and self-reflective learning process in which students solve unstructured, real-life problems (Hung et al., 2008). In PBL, the instructor plays the role of a facilitator rather than a deliverer of information, and students form small groups to generate problems on their own, seek solutions, and experience interdisciplinary learning that integrates what they have learned (De Graaf and Kolmos, 2003). The importance of PBL as an innovative teaching method has been emphasized, and many previous studies have shown that it helps improve students’ competencies, such as self-directed learning, problem-solving, critical thinking, and communication ability (Kim, 2017; Chang et al., 2022; Yun, 2024).

However, previous studies in South Korea focused on the quantitative changes experienced by students participating in PBL. For instance, Nam and Yoon (2016) examined 57 students’ changes after participating in a PBL course via pre- and post-tests of self-regulated learning and problem-solving ability. Chang et al. (2022) also examined the effect of PBL on 34 students by investigating changes in self-directed learning, problem-solving ability, and critical thinking disposition. Other studies conducted a meta-analysis to examine the learning effects of PBL (Park and Woo, 2017; Park et al., 2023). While these studies clearly show the effects of PBL on fostering various abilities and dispositions, the quantitative approach does not illustrate how these changes occur. Hence, exploring the specific experiences and processes of PBL that foster learning and change would be meaningful in devising new instructional methods for students to cultivate their generic skills.

Therefore, the present study aims to explore how university students subjectively experience PBL and to identify how these experiences are related to their generic skills, defined here as cross-cutting competencies acquired beyond domain-specific knowledge. For this purpose, the researchers analyzed reflection papers submitted by students who were taking a PBL course as a major-related elective course. The document data were collected from three PBL classes offered at a four-year university in Seoul, Korea, between 2022 and 2023. The data was analyzed using the thematic analysis by Braun and Clarke (2006), which is useful for identifying patterns in the collected data and exploring research topics from various angles. Hence, the current study applied an inductive approach to derive recurring themes and patterns from students’ reflections on PBL classes. Based on the analysis, the study intended to provide a more in-depth understanding of how students experience PBL and extend the discussion on what aspects relate to students’ skills.

## 2 Literature review

### 2.1 Generic skills in higher education

Generic skills are abilities that can be utilized across different contexts, extending beyond the confines of specific fields or disciplines (Barrie, 2006). There are various terms in the literature used to refer to generic skills (Zouaoui et al., 2024), namely transferable skills (Hoidn and Kärkkäinen, 2014), core competencies (Virtanen and Tynjälä, 2019), soft, transversal, or employability competences (Pažur Aničić et al., 2023), or non-academic skills (Aliu and Aigbavboa, 2023). Generic skills have been examined in many different countries as one of the key factors impacting students’ employability that help them be prepared for an adaptive transition from school to work (Ebaid, 2021; Aliu and Aigbavboa, 2023; Iqbal et al., 2023; Pažur Aničić et al., 2023).

There is no consensus or an exhaustive list of skills that define generic skills. A scoping review by Zouaoui et al. (2024) proposed a comprehensive category including personal, idea- and object-related, interpersonal, and community-related skills. Lee and Lee (2022) investigated generic skills related to university students’ significant learning experiences and deduced comprehensive thinking skills, information utilization skills, interpersonal skills, and personal attributes. Aliu and Aigbavboa (2023) examined the list of skills from existing literature and organized 15 categories of skills, including adaptability skills, entrepreneurship skills, and willingness to learn.

Although there is no fixed set of skills, studies have shown that cultivating these transferable, non-academic skills is required in the world of work. A study investigating generic skills that employers expect from their employees found that most employers require skills such as leadership and management, critical thinking or problem-solving skills, and social or teamwork skills (Alias et al., 2013). In another study, in which employers rated the importance of certain technical skills and generic skills of accounting graduates, it was found that only 8 out of 12 technical skills were identified as important, while all 12 generic skills were rated as important (Ebaid, 2021). As generic skills grow increasingly vital for students in higher education, particularly for a successful transition from university to the workplace, identifying effective strategies to cultivate these competencies has become essential.

### 2.2 Problem-based learning as a means to foster generic skills

Problem-based learning (PBL) is an innovative pedagogical approach that involves students in identifying and solving authentic and unstructured problems through teamwork (Hmelo-Silver, 2004; Yew and Goh, 2016). The learning principle underlying PBL is that learning should be a process that is constructive, self-directed, collaborative, and contextual (Dolmans et al., 2005). To create a learning environment for students to engage in such a learning process, students should encounter more complex and real-life problems that are ill-structured and open-ended (Dolmans et al., 2005). Also, students engage in self-directed and collaborative learning processes to generate solutions to the identified problems (Yew and Goh, 2016).

In a meta-analysis on the effectiveness of PBL, Strobel and Van Barneveld (2009) found that PBL is effective in retaining long-term knowledge, producing performance or skill-based learning outcomes, and acquiring integrative knowledge and skills. PBL was also found to be effective in enhancing students’ self-directed learning, problem-solving, and critical thinking (Chang et al., 2022), satisfaction with learning (Kim, 2017), and self-determined motivation (Yun, 2024).

PBL is an effective way to promote various skill sets that enhance students’ employability, in turn, helping students navigate through the rapidly changing world of work (Hoidn and Kärkkäinen, 2014). However, it is unclear as to what specific aspects of PBL contribute to students’ learning and skill development (Yew and Goh, 2016). Hence, it would be important to take a closer look at what students experience in the process of PBL to identify factors that promote their growth.

## 3 Methods

### 3.1 Research design

This study employed a qualitative research design using students’ reflection papers as documents for the analysis. Documents are written texts that take various forms such as notes, case reports, diaries, and letters (Wolff, 2004). Documents used in research can either be solicited specifically for the research or unsolicited (Flick, 2019). According to Lincoln and Guba (1985), documents that are produced during personal activities, which can be interpreted within a specific context, can be used as data in research. In the present study, students’ reflection papers collected in PBL courses were used as documents. To explore students’ subjective experience of PBL, thematic analysis was applied to identify, categorize, and interpret recurring patterns and themes (Braun and Clarke, 2006).

### 3.2 Characteristics of data

The document data used in this study are students’ reflection papers collected in three PBL classes, namely two Social Survey Method classes and one Educational Assessment class, offered at a 4-year university in Seoul, Korea, between 2022 and 2023. Before designing the PBL courses, the authors first participated in the orientation of PBL from the Center for Teaching and Learning (CTL), and received individual consultation for each class from the research professor at CTL, a Ph. D. in Educational Technology, to make sure that the PBL elements were well incorporated into each course. The corresponding author instructed all three courses.

Each course was designed with two PBL modules, one three-week module before the midterm and another four-week module after the midterm. After completing the two modules, students were asked to submit their reflection papers guided by open-ended questions. The guiding questions include “What did you experience while engaging in PBL?” and “What strengths or skills did you use or newly discover?” Each reflection paper was one to two pages long.

A total of 58 reflection papers were used in the final analysis, 27 from Educational Assessment in 2023, 18 from Social Survey Method in 2023, and 13 from Social Survey Method in 2022. The characteristics of the students who participated in the course and submitted the papers are presented in Table 1.

**Table 1:** Characteristics of students who submitted the reflection papers.

	2022–2 Social survey method	2023–1 Educational assessment	2023–2 Social survey method	Total Frequency (%)	
Sex					
Men	3	5	8	16	(27.59)
Women	10	22	10	42	(72.41)
Grade					
Sophomore	-	-	4	4	(6.90)
Junior	4	27	7	38	(65.52)
Senior	9	-	7	16	(27.59)
Major					
Humanities and social science	13	7	18	38	(65.52)
Arts and kinesiology	-	17	-	17	(29.31)
Science	-	3	-	3	(3.17)
Total	13	27	18	58	(100.00)

### 3.3 Data collection

The reflection papers were collected at the end of each PBL module as a required course assignment. The papers were submitted via the university’s Learning Management System (LMS). Since the reflection papers were a part of course participation, an IRB review was conducted before using the papers as document data for research. Noting that the papers submitted via LMS could be downloaded, excluding any personally identifiable information, and that the courses were already completed with final grades assigned and course evaluation conducted, an IRB waiver was obtained (SU-2024-E-010). After the ethical review, students’ responses to the open-ended questions were downloaded into an Excel file for the analysis.

### 3.4 Analysis

The collected document data was analyzed by applying the thematic analysis of Braun and Clarke (2006). The thematic analysis provides a flexible way to identify patterns and explore research topics from various angles (Braun and Clarke, 2006), so it was applied to the current study to identify recurring themes and patterns from students’ reflection papers to explore their experience of engaging in PBL. While this study aimed to explore students’ experiences with PBL in relation to the development of generic skills, the authors refrained from creating a predefined codebook of generic skills to categorize the data. Instead, the authors implemented an inductive method to discover themes from the data, as suggested by Braun and Clarke (2021). The authors followed the six steps of analysis offered by Braun and Clarke (2006). First, each author tried to become familiar with the documents by repeatedly reading them. To understand the context of the data, the authors also reviewed the course syllabus and students’ anonymous course evaluations submitted at the end of the semester. Second, the authors individually created codes for the data and compared them. To capture the meaning and the context of the document, the authors considered sentences or paragraphs as the unit of coding (Saldaña, 2021). When the authors’ codes were in discord or when certain excerpts were coded by only one of the authors, the authors went back to the original text of the reflection papers to reexamine and discuss the context. The codes that both authors agreed on and had ample supporting excerpts remained for the analysis. Third, the codes were organized into potential themes, which were then analyzed for their relationships, leading to the development of themes and sub-themes. Fourth, codes and the original texts were revisited to examine whether the themes and sub-themes appropriately captured the essence of the data. Fifth, the authors reexamined the names of the themes and sub-themes so that the names succinctly capture the core ideas. Finally, the process of the entire analysis was reviewed, and the results were written.

### 3.5 Trustworthiness

Guidelines suggested by Curtin and Fossey (2007) were followed for trustworthiness. First, a thorough description of the document characteristics, document collection, and analysis process are provided to ensure the clarity of the research process. Second, researcher triangulation was implemented, in which the authors, who have experience in conducting qualitative research, analyzed the document data separately before discussing the initial codes and analysis. Third, to check for transferability, the themes and the results were audited by two researchers experienced in qualitative research and PBL. One was a Ph.D. in educational administration, and the other was a Ph.D. in English linguistics, both with more than 5 years of teaching experience as professors at a four-year university in Korea. The auditors examined whether the findings could be applied to their students’ experiences of PBL. Finally, to ensure reflexivity, the authors took notes of their previously held assumptions and reflected on them during analysis so that the authors’ perspectives did not hinder capturing the essence of students’ experiences.

## 4 Findings

**Figure 1:**
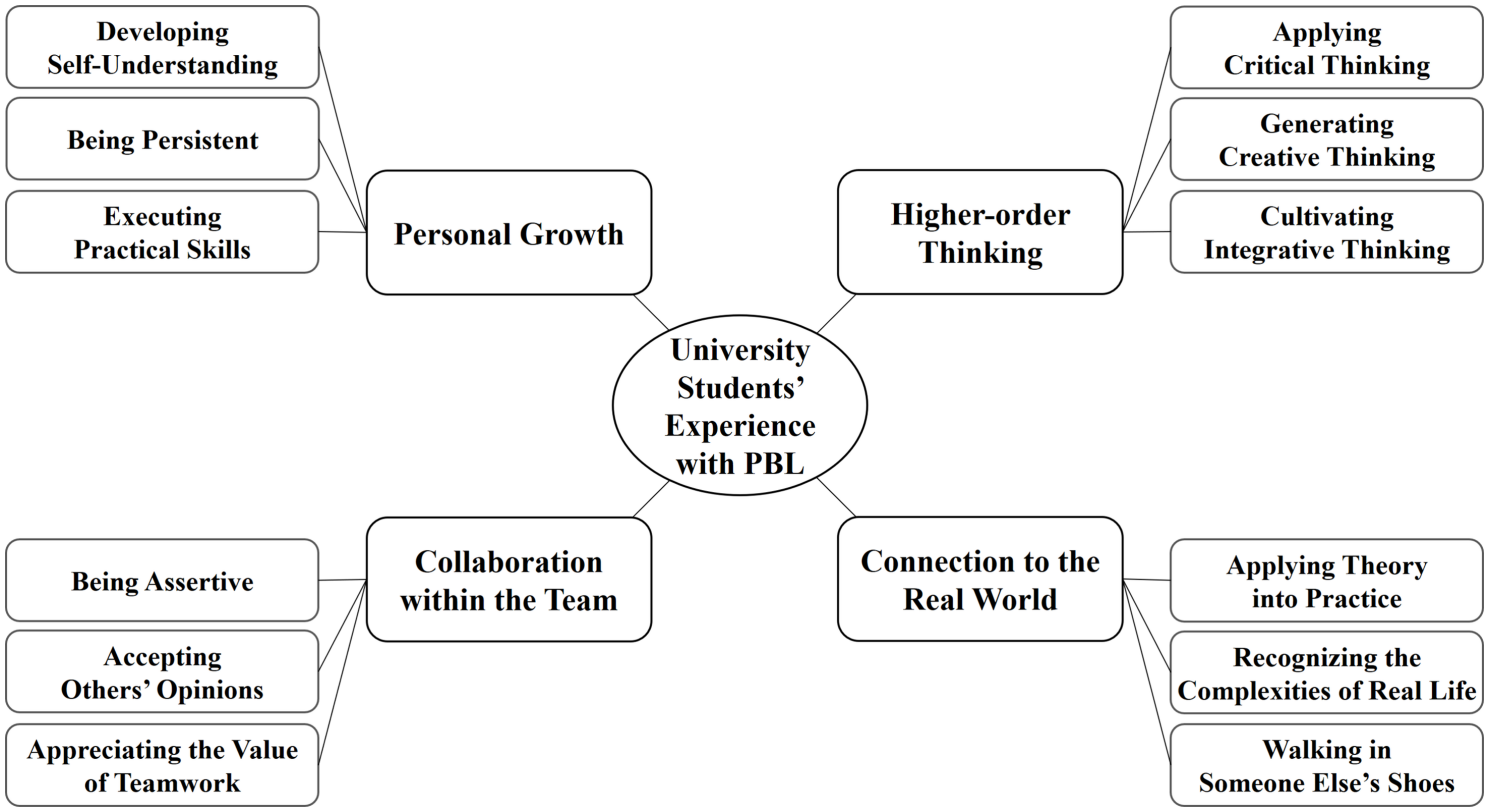
Themes and sub-themes of students’ PBL experience.

The analysis derived four themes from students’ experience with PBL: collaboration within the team, personal growth, higher-order thinking, and connection to the real world. These themes consisted of 12 sub-themes highlighting the accompanying generic skills illustrated in Figure 1.

### 4.1 Collaboration within the team

Working within the team had significant meaning to all students. They had had previous experiences working in teams, but working in PBL teams was experienced differently from other team projects. The sub-themes include being assertive, accepting others’ opinions, and appreciating the value of teamwork.

Most of all, students reflected on their experience of being assertive. Sharing one’s opinion in teamwork seems to be a natural and unremarkable aspect. However, it was notable that students were previously reluctant to share their ideas actively because they were not confident in themselves. They were also less assertive in giving feedback because they viewed it as judging others, diminishing others’ efforts, or hurting others’ feelings. In the PBL process, in which team members worked together with respect and empathy to reach the same goal, students took the initiative to express themselves.

I thought that giving negative feedback on others’ results would hurt their feelings, but my team members freely gave and received positive and negative feedback. Through this, our team’s results improved significantly. Through this, I learned that to achieve the team’s goals more effectively, sometimes it is necessary to have the courage to give objective and realistic feedback [Participant 38].

The experience that went hand in hand with being assertive was accepting others’ opinions. Receiving others’ feedback was identified as a valuable experience because each team could produce better outcomes by reflecting on various viewpoints. Students reported that in other team projects, the team members were working on the *same* project, but not actually working *together*. They simply divided tasks, such as data searching, making presentation material, and doing the presentation, and worked separately on each given part. There was not much room for sharing ideas and taking in different perspectives. Hence, students had not fully experienced the value of refining their work by inviting others’ ideas. With PBL, on the other hand, students spent ample time together to generate ideas, and in that process, they learned to respect and accept others’ opinions.

Before doing this PBL, I did not prefer receiving feedback. Rather than respecting others’ opinions, I thought my opinion was more important. Now I learned that when I accept others’ opinions, I can achieve better results and resolve problems much faster. Now that I have learned to accept others’ feedback to complement weaknesses and highlight strengths, I am more confident in sharing my ideas and getting feedback from others [Participant 27].

In PBL teams, all team members contribute to the work together. Team members’ ideas, efforts, skills, team cohesion, and respect for one another came together to produce better outcomes that individuals could not have achieved. This was a notable experience for many students because they recognized the value of teamwork. Some students even used the term “power of collective intelligence” to describe their experiences of how their teamwork produced better results. Not only did they strive for better results as a team, but the membership helped students learn from one another and become more productive.

The PBL module allowed me to recognize my shortcomings, and working with my teammates helped me improve them. I learned a lot from my teammates as I experienced that better results could be achieved by talking to them and giving and receiving feedback. I experienced that teamwork and cooperation are important because teammates share different perspectives and new problem-solving methods. It was better than doing things alone [Participant 25].

As a team, students generated new ideas by expressing their opinions and respecting others’ perspectives. They could also pursue higher-quality work as a team because they learned to appreciate teamwork. Such experiences led to students’ enhanced ability to collaborate within a team.

### 4.2 Personal growth

Students reflected on their personal growth in the process of participating in PBL. The sub-themes discovered from the data include developing self-understanding, being persistent, and executing practical skills. First, many students extended their self-understanding by finding new strengths or areas of growth and changing their previously held perception of themselves. Various newly found strengths were mentioned in the reflection papers, including management skills, creativity, meticulousness, and so on, but the most notably mentioned aspect was interpersonal skills. This strength was closely related to how students changed their views of themselves. Many students previously identified themselves as being introverted, people-fearing, or too sensitive. They were reluctant to participate in PBL or were unsure whether they could succeed in the course. However, as the PBL progressed and the team cohesion increased, these students found that they enjoyed working with others and could express themselves without fearing others’ judgment.

The fear of people disappeared. I always had difficulty with relationships with people, so I used to work passively on team projects. However, through the PBL activity, I was able to break down that wall that had hindered relationships… I am introverted, so I could not express my honest opinions before, but my team members made me feel comfortable, and for the first time, I could carry out the group project without feeling pressured… It was rewarding because I was able to develop the ability to cooperate effectively with others [Participant 12].

Students also reported that they learned to persist through the challenges. The PBL process was experienced as ambiguous with no clear answers. Some students felt anxious about not being certain whether they were going in the right direction. However, they repeatedly tried and persevered to reach their own goals.

I wanted to be of some help to the team. So I repeatedly solved the example problems in the textbook until I became familiar with the concepts. I persisted, and I was able to improve my ability to apply the concepts to other problems and find solutions myself [Participant 42].

Students also reflected on improving practical skills, such as time management, prioritizing tasks, allocating roles, data searching strategies, organizing information, and writing reports. Time management, prioritization skills, and allocating roles were improved mainly to “proceed with activities efficiently and meet deadlines by eliminating unnecessary processes” [Participant 40]. Students managed time and the given tasks so that all team members could contribute to achieving the team goal. Data searching strategies were also related to goal orientation. When learning was focused on going through a given textbook through lectures, students merely followed the curriculum and became passive learners. However, with PBL activities, students actively searched for and selected “relevant and reliable data” [Participant 35] that could be applied to their project. This was perceived as a meaningful experience because students gained the ability to navigate through a vast amount of resources and make decisions to identify information that was necessary for achieving their goals.

When analyzing the research problem, I could conduct efficient data research by setting keywords appropriately while investigating through many different references… I was able to increase my understanding of the research methodology [Participant 37].

By implementing more strategic data searching, students also learned to organize data and write it into formal reports. Students reported that this process was meaningful because it was not simply producing a piece of writing for an assignment, but a way of integrating and synthesizing what they had learned. Students viewed their final PBL reports as being more informative, persuasive, and meaningful compared to other assignments they had worked on before.

In sum, students reported that they experienced growth on a personal level. They gained a new understanding of themselves, learned to persist through challenges, and enhanced their skills in managing their projects. These experiences occurred in the context of cohesive teamwork and self-directed learning.

### 4.3 Higher-order thinking

Students reflected on incorporating different thinking skills while searching for problems and devising ways to resolve them. This theme includes applying critical thinking, generating creative thinking, and cultivating integrative thinking as sub-themes. Most of all, critical thinking was often addressed. Students reviewed and critically analyzed their ideas to find the best answers.

When I lost my sense of direction or when my judgment became clouded, I never stopped thinking critically. Even when things seemed okay or could be overlooked, I constantly revised and supplemented our work by thinking about how I would feel if I were on the receiving end of the survey [Participant 58].

Students also reflected on enhancing creative thinking skills. Creative thinking was possible because students were active learners in the PBL process. They were not merely taking in the information provided to them but identifying problems and generating new ideas to solve them. They were also engaged in active discussions in which different perspectives were shared, leading students to think out of the box. Active, autonomous, interactive participation allowed students to engage in creative thinking.

I used to simply memorize the course contents, but in this activity, I did a lot of thinking on my own… I tried to utilize knowledge rather than just memorize it. It naturally led to creative ideas as I thought about new ways to find commonalities, combine concepts, and create cases to apply the concepts [Participant 22].

Furthermore, because each team consisted of members from different majors, each member brought a new perspective to the problem. Even when working on the same task, members approached the problems from various angles. Students learned to appreciate these differences and expanded their views by integrating different perspectives.

When trying to combine aspects of my major with other majors, I think I began to think out of the box… It was eye-opening to see that my major, which I thought was a narrow field, could be applied to completely different fields. The activity made me think more broadly about my career path as well [Participant 19].

Students reported that through the PBL process, they improved their critical, creative, and integrative thinking skills. These thinking skills were used not only to solve their PBL problem but also to gain a deeper understanding of the course material and expand their knowledge.

### 4.4 Connection to the real world

The purpose of PBL is to help learners identify and solve authentic, ill-structured, complex problems that we encounter in real life. As such, most students reflected on building a connection to the real world by applying theories into practice, recognizing the complexities of real life, and walking in someone else’s shoes.

Most students addressed how they applied the theories and concepts they had learned in the textbooks to real-life situations. The theories were not limited to those they learned in the PBL course but included various materials they had obtained in other courses as well. Students reported that by participating in PBL activities, they were able to apply what they had learned and experience how their knowledge could be used in practice.

Based on what we learned, we directly collected data, refined it, created variables, and figured out which analysis or refinement to use to solve the problem… By applying the knowledge that the professor taught us, I felt that I could deeply internalize the understanding I gained in class [Participant 34].

While students tried to integrate their classroom knowledge to reach out and work through real-world obstacles, they also faced the complexities of reality. In theory, concepts seemed simple and obvious. When observing others working in society, their tasks seemed fairly straightforward. However, when students tried to solve real-life problems, they realized that there were so many little details to consider. It was far more complex and complicated than they had expected.

When I learned the theory, I had a superficial and abstract understanding of the principles. However, as I tackled actual problems, I encountered various difficulties and adversities… I realized that a lot of detailed planning and effort was needed… I had not realized that so much care was necessary [Participant 14].

Engaging in PBL activity meant that students had to take on different roles necessary to solve the given problems. For instance, in the Social Survey Method class, students were the researchers investigating social phenomena. In the Educational Assessment class, students were the teachers and educators trying to evaluate learners. By taking on these different roles to identify the problems and devise ways to solve them, students had the chance to walk in someone else’s shoes. They recognized the work and effort that different people put into their work and learned to appreciate it. It should also be noted that students not only identified with the roles they took, namely social researcher or educator, but also considered the position of the research participant, the general public receiving the research results, and the learners being evaluated. Participating in PBL activities helped students take a step back from their own perspective and consider others’ viewpoints from various angles.

I got to think from the teacher’s perspective. I became a teacher, making questions for the first time, and realized that creating a single question takes a lot more thought and agony than I expected. Also, I realized that as a teacher, I had to be fully aware of all the content I teach to learners, consider which parts are more important, and what learners must understand. It was an unfamiliar and difficult process, but I was able to gain a lot from the hardships, so I think it was a fun and rewarding experience [Participant 1].

By engaging in PBL activities, students were actively making connections to the world to which they would be heading. They were using the contents they learned in class to understand and tackle real-life problems. Through experiencing authentic tasks, they recognized the complexities of the actual world and learned to consider the different perspectives of those involved in the phenomena.

## 5 Discussion and conclusion

### 5.1 Findings and implications

The present study aimed to investigate university students’ experience of PBL and to explore how these experiences are related to generic skills applicable in multiple contexts. To derive meaningful aspects of students’ experiences, 58 reflection papers were collected from students who participated in a PBL course in 2022 and 2023, at a four-year university in Seoul, Korea. The thematic analysis was used to explore the collected documents. As a result, four themes were derived from the data: collaboration within the team, personal growth, higher-order thinking, and connection to the real world.

The first theme, collaboration within the team, reflects the essence of PBL, which is designed to help learners be effective collaborators who can exchange ideas openly and work toward a common goal (Hmelo-Silver, 2004). The findings of the study uncovered that students become more assertive in expressing their ideas and also accepting of others’ opinions when engaging in the PBL process. These experiences of conveying ideas in discussions and respectfully listening to others are related to interpersonal skills that are enhanced through group work (Ainiyah et al., 2022). They are also associated with communication skills, which involve proficiency in attentive listening, verbal expression, and empathy towards others (Aliu and Aigbavboa, 2023). Such communication skills have been found to serve as a key factor contributing to effective teamwork (Devece et al., 2014). Hence, interpersonal skills, communication skills, and teamwork are closely related, influencing one another in the process of PBL. Moreover, the findings of this study show that students learned to recognize and appreciate working as a team. This reflects teamwork skills, referred to as working effectively in groups and striving for better performance (Aliu and Aigbavboa, 2023). Students learning to value teamwork is particularly meaningful in the Korean context because the intense competition for university admission has fostered a highly competitive academic culture (Lee and Jeon, 2022). As reflected in students’ papers, students were previously reluctant to give or receive feedback because it was perceived as judgment. They also did not appreciate others’ opinions because their own opinions mattered more. However, in PBL, students experienced that working together as a team was more meaningful and valuable in that they learned more and produced better outcomes. While some studies, such as de Prada Creo et al. (2021), focused on the acquisition of teamwork skills through extra-curricular activities, such as sports or volunteering, this study showed that students can foster their teamwork skills while participating in a regular course.

Personal growth was the second theme derived from students’ experiences. Since PBL was a longer-term project compared to other course assignments, and required active involvement and interaction with team members, students were able to take the time to monitor themselves and identify aspects that contributed to the learning process. The sub-themes included developing self-understanding, which encompassed finding new strengths or weaknesses and gaining new perspectives of oneself; demonstrating persistence in the face of challenge; and learning to execute various practical skills such as time management, data searching, organizing information, and writing reports. According to students’ reports, personal growth was made possible by the reciprocal respect and support among team members, aligning with the previous research showing that supportive social relationships facilitate personal growth (Lee et al., 2018). Within the team, students engaged in self-reflection to gain a deeper understanding of themselves, and such self-reflection can be interpreted as relating to personal competence, defined as a positive attitude toward self-development and learning (Braun and Leidner, 2009). Also, students strived to persist when facing challenges because they wanted to contribute to the team, indicating their skill of resilience and coping (Zouaoui et al., 2024). Using practical skills to manage team meetings and projects involves planning and organizational skills (Zouaoui et al., 2024), as well as the use of self-management, ability of written communication, and information processing (K-CESA, 2024). Students were able to experience personal growth and foster various skills while engaging in teamwork.

The third theme, higher-order thinking, encompasses critical thinking, creative thinking, and integrative thinking. Numerous studies have found that PBL is effective in enhancing learners’ critical thinking (Liu and Pásztor, 2022; Anggraeni et al., 2023) and creative thinking (Widiastuti et al., 2023). Also, since PBL aims to help learners build a comprehensive and flexible body of knowledge (Hmelo-Silver, 2004), students actively shared their knowledge and experiences with team members to integrate perspectives and theories from different academic disciplines. Such thinking skills are commonly addressed as transferable skills in literature and found to be associated with various experiences such as independent learning, collaborative learning, and application of knowledge to real-life situations (Lee and Lee, 2022). Higher-order thinking skills are related to the cognitive process of utilizing knowledge that is essential for students to address the challenges of the rapidly changing society (Kang et al., 2010).

Finally, connection to the real world was the fourth theme, and it involved applying theories into practice and recognizing the complexities of real life. In the PBL process, students encounter complex and unstructured real-life problems to which there are no clear solutions (Dolmans et al., 2005). Thus, students have to integrate what they have learned thus far to solve the multifaceted problems. These experiences reflect the use of knowledge processing and the ability to use and apply previously learned knowledge and skills (Braun and Leidner, 2009). Another sub-theme, walking in someone else’s shoes, reflected students’ experience of understanding, empathizing, and appreciating the roles and work of others in society. Students reported having the opportunity to adopt others’ perspectives and gain insight into how they feel and perceive things. This experience can be understood as a reflection of ethics of care, encompassing empathy, compassion, and courtesy (Zouaoui et al., 2024). Rather than mechanically solving a given problem, students learn to consider the perspectives of those directly affected by the problem in real-life contexts and develop a sense of empathy through engaging in the PBL process.

It should be noted that collaboration within the team was the foundational aspect that allowed for other themes to emerge. That is, students’ personal growth, higher-order thinking, and building connections to the real world occurred in the context of teamwork. Team members’ support, respect, and interactions not only fostered collaboration but also other aspects of learning. Yew and Goh (2016) investigated previous studies on the process of PBL and found that studies either focus on collaborative interactions or individual self-directed learning as a major aspect of PBL that impacts students’ learning achievement, but opined that it is still inconclusive whether collaboration or self-directedness leads to learning. However, in the current study, collaborative interactions were the major element. Students willingly searched for information, actively organized and expressed their thoughts, persevered through challenges, and strived for higher-quality work because they were working in a team that was meaningful and important to them. Yew and Goh's (2016) study merely states collaborative interactions but does not clearly describe what kind of collaborative interactions. In the current study, the team was something that the students wanted to contribute to, were supported by it, provided rewarding learning experiences, and respected and complimented their work. Hence, it is not just the collaboration that matters, but what kind of collaboration is being generated within a team that matters.

### 5.2 Limitations and directions for future study

The current study has several limitations. First, the study analyzed the document data, which allowed to capture many students’ experiences, but should be complemented by in-depth individual or group interviews. In the reflection papers, some of the words and statements stood out as meaningful experiences, such as gaining confidence, but the writing itself did not fully describe the experience, and so was excluded from the current analysis. Interviews may help gain a more comprehensive understanding of the student’s experiences. Second, since the current study intended to discover themes from the documents, the codes and themes were generated from the data, and they were not directly matched to a list of generic skills. To understand and link the PBL experiences with specific generic skills, structured questionnaires or deductive analysis may be used for future study. Third, the current study investigated what students experienced in a single-semester PBL course, but it would also be important to follow up and investigate whether their experiences have a longer-term impact on various areas of their lives, such as learning in other courses, engaging in different team projects, and career preparation. Fourth, the PBL courses in which the reflection papers were collected were conducted by the corresponding author and maintained certain consistency. However, the management and operation of PBL and the course contents may have different impacts on the PBL experience of students. Hence, future studies should consider comparing PBL courses with various course materials or instructor styles.

Despite such limitations, the current study provides meaningful insight into university students’ experience of PBL in the Korean context and how these experiences may be related to the development of generic skills. Based on the findings, higher education may consider incorporating PBL courses to help students experience meaningful learning experiences and foster various skills that can be transferred to different aspects of life.

## Data Availability

The raw data supporting the conclusions of this article will be made available by the authors, without undue reservation.
